# Depiction of pneumothoraces in a large animal model using x-ray dark-field radiography

**DOI:** 10.1038/s41598-018-20985-y

**Published:** 2018-02-08

**Authors:** Katharina Hellbach, Andrea Baehr, Fabio De Marco, Konstantin Willer, Lukas B. Gromann, Julia Herzen, Michaela Dmochewitz, Sigrid Auweter, Alexander A. Fingerle, Peter B. Noël, Ernst J. Rummeny, Andre Yaroshenko, Hanns-Ingo Maack, Thomas Pralow, Hendrik van der Heijden, Nataly Wieberneit, Roland Proksa, Thomas Koehler, Karsten Rindt, Tobias J. Schroeter, Juergen Mohr, Fabian Bamberg, Birgit Ertl-Wagner, Franz Pfeiffer, Maximilian F. Reiser

**Affiliations:** 1 Department of Radiology, University Hospital, LMU Munich, 81377 Munich, Germany; 2 Chair for Molecular Animal Breeding and Biotechnology, Ludwig-Maximilians-University Munich, Munich, 85764 Oberschleißheim Germany; 3 Chair of Biomedical Physics & Munich School of BioEngineering, Technical University of Munich, Munich, 85748 Garching Germany; 4 Department of Diagnostic and Interventional Radiology, Technical University of Munich, 81675 Munich, Germany; 5 Philips Medical Systems DMC GmbH, 22335 Hamburg, Germany; 6 Philips GmbH Innovative Technologies, Research Laboratories, 22335 Hamburg, Germany; 7 Institute for Advanced Study, Technical University of Munich, 85748 Garching, Germany; 8 Institute of Microstructure Technology, Karlsruhe Institute of Technology (KIT), 76344 Eggenstein-Leopoldshafen, Germany; 9 German Center for Lung Research (DZL), Comprehensive Pneumology Center (CPC-M), Helmholtz Zentrum Munich, 81377 Munich, Germany

## Abstract

The aim of this study was to assess the diagnostic value of x-ray dark-field radiography to detect pneumothoraces in a pig model. Eight pigs were imaged with an experimental grating-based large-animal dark-field scanner before and after induction of a unilateral pneumothorax. Image contrast-to-noise ratios between lung tissue and the air-filled pleural cavity were quantified for transmission and dark-field radiograms. The projected area in the object plane of the inflated lung was measured in dark-field images to quantify the collapse of lung parenchyma due to a pneumothorax. Means and standard deviations for lung sizes and signal intensities from dark-field and transmission images were tested for statistical significance using Student’s two-tailed *t*-test for paired samples. The contrast-to-noise ratio between the air-filled pleural space of lateral pneumothoraces and lung tissue was significantly higher in the dark-field (3.65 ± 0.9) than in the transmission images (1.13 ± 1.1; *p = *0.002). In case of dorsally located pneumothoraces, a significant decrease (−20.5%; *p* > 0.0001) in the projected area of inflated lung parenchyma was found after a pneumothorax was induced. Therefore, the detection of pneumothoraces in x-ray dark-field radiography was facilitated compared to transmission imaging in a large animal model.

## Introduction

Several studies on pulmonary diseases in mouse models have demonstrated dark-field radiography of the lung to improve the diagnosis of pulmonary emphysema^1–3^, fibrosis^4^, bronchopulmonary dysplasia^5^, lung cancer^6^, and pneumothoraces^7^. Investigation of animal models with chest dimensions comparable to humans is an essential step toward the potential *in vivo* implementation of this new imaging modality in a clinical setting. There are major challenges that have so far hindered the translation of the murine experiments into larger animal models and subsequently into a potential human clinical application. These include the radiation dose, the capability to penetrate larger objects comparable to humans, and the development of a field-of-view equivalent to the human chest without compromising image quality or prolonging data acquisition time beyond the breath-hold limit. These challenges have recently been largely overcome and a prototype dark-field large animal scanner that allows dark-field chest radiography with a clinically applicable setup has been developed^8^.

A pneumothorax is characterized by the presence of air in the pleural cavity that leads to a collapse of the affected lung^9^. Causes may be iatrogenic, posttraumatic, or idiopathic; pneumothoraces often require immediate treatment in order to avoid severe health consequences^10^. Medical imaging is usually required to reliably diagnose a pneumothorax, as clinical signs are often unspecific and do not reflect the amount of air in the pleural cavity^11^. Chest computed tomography serves as the standard of reference in diagnosing and sizing pneumothoraces^12^. However, its use is limited in clinical practice due to relatively high cost and radiation dose, as well as inconvenience for critically ill patients^13^. As conventional chest radiographs are readily available, cost- and time-effective, they are usually the first test ordered for a suspected pneumothorax^14^, although there are limitations resulting in a considerable number of missed pneumothoraces^15–17^. Due to the low density of lung tissue, the difference in attenuation between air and lung is very small and can be easily overlooked. In fact, when physicians other than radiologists interpret radiographs, up to 76% of pneumothoraces are missed^18^. Comparatively high rates of misdiagnosed pneumothoraces have also been reported for inexperienced radiologists^19^.

If the patient is in an upright position while the radiograph is taken, the air in the pleural cavity usually collects around the lung apices where a thin, sharp line, which might be masked by the rib shadow, indicates the edge of the lung. However, in critically ill patients, chest x-rays need to be acquired in the supine position with air accumulating in the ventral pleural space^20^ resulting in an overall sensitivity of only 50%^21^. Tubes and lines can further reduce the diagnostic sensitivity^22^.

Dark-field projection radiography may potentially solve these diagnostic problems. In a small animal model, murine lungs with pneumothoraces showed a sharp contrast difference between the strongly scattering lung parenchyma (strong dark-field signal), and the air filled pleural space (no measurable dark-field signal)^7^. At this point, it is of immense clinical interest to transfer the information provided by the small animal studies to the now available large-object chest x-ray dark-field prototype, to test for the applicability of dark-field imaging for objects of human size. We therefore aimed to evaluate the added clinical value of dark-field imaging for the diagnosis of pneumothoraces in pigs with artificially induced, unilateral pneumothoraces – the first disease model ever tested using a large animal x-ray dark-field radiography device.

## Results

Signal analysis of lung-to-pneumothorax CNRs in the transmission and dark-field modalities did not reveal a statistically significant difference between *ex vivo* with *in vivo* lungs [*p*(Transmission) = 0.36; *p*(Dark-field) = 0.17].

### Lateral pneumothoraces – visual and quantitative analyses

**Figure 1:**
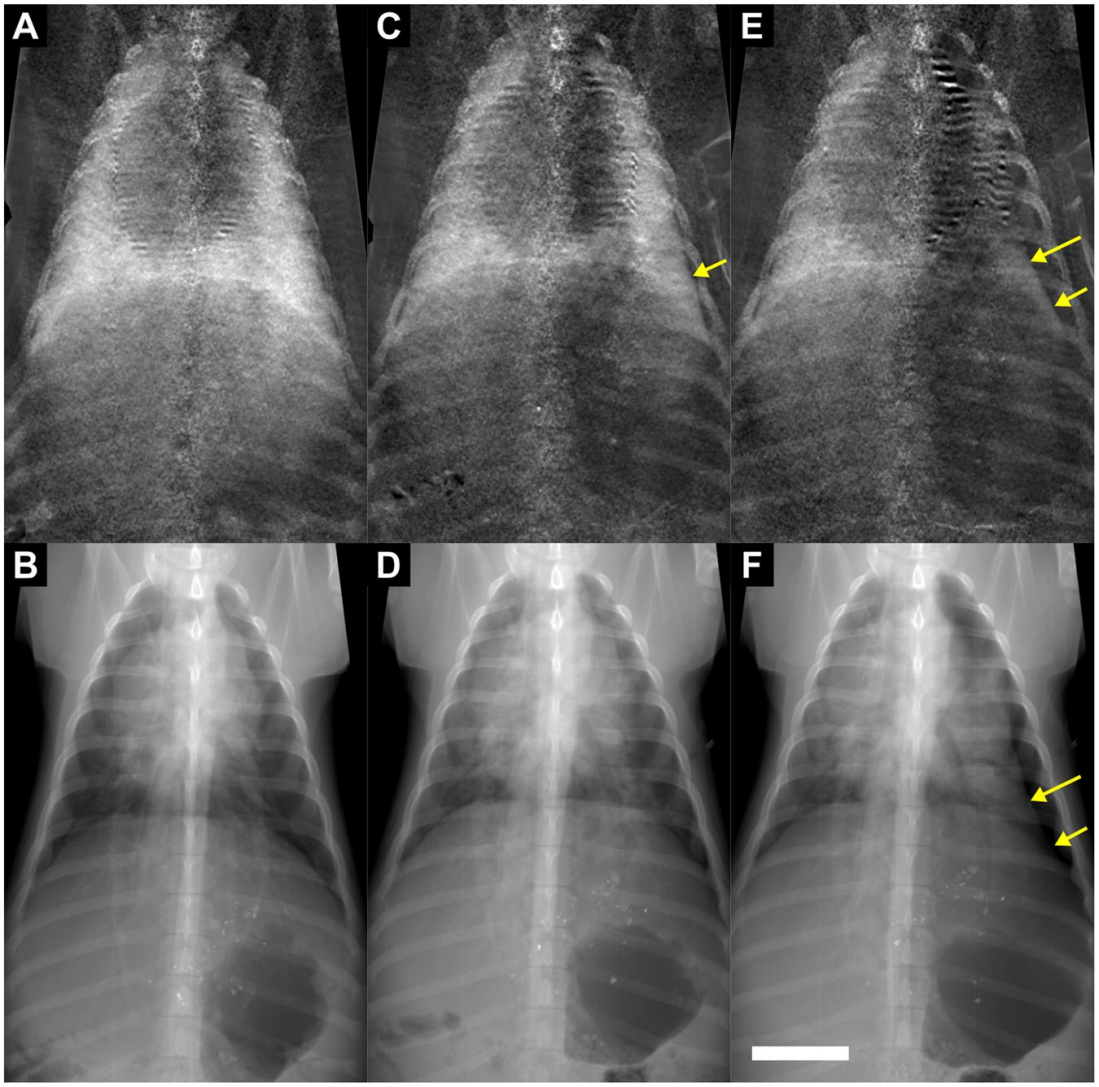
Pneumothoraces of different sizes depicted in x-ray dark-field and transmission images.Chest radiographs of the same pig (*in vivo*) just before (**A**,**B**) and after application of 250 ml air (**C**,**D**) and 500 ml air (**E**,**F**) in the left pleural space, comparing transmission images (lower row) with the corresponding dark-field images (upper row). A pneumothorax is indicated in dark-field images as a dark area adjacent to the rib cage on the left side (arrows). After application of 250 ml of air, a small pneumothorax was only visible in the dark-field image, indicated by a discrete widening of the left pleural space. The white scale bar is approximately 5 cm.

**Figure 2:**
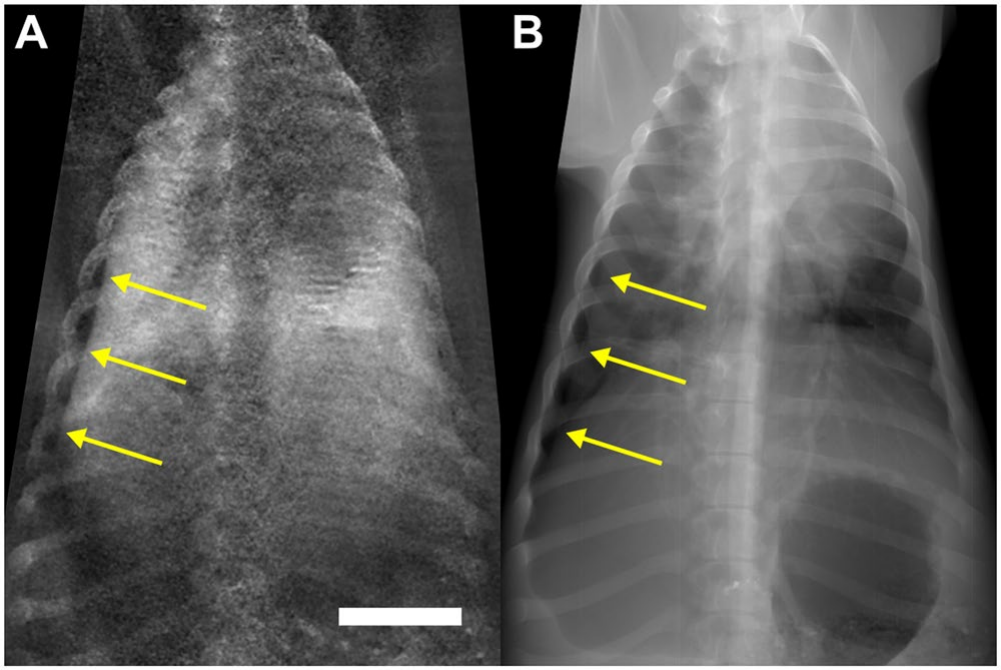
Unilateral pneumothorax depicted in x-ray dark-field and transmission images.Chest radiographs of a pig (*in vivo*) after a pneumothorax (arrows) was induced on the right side. The transmission image (**B**) can be compared directly to the corresponding dark-field image (**A**). The white scale bar is approximately 5 cm.

As proven by CT, all experimental animals had developed a unilateral pneumothorax. All three pigs measured *ex vivo* and three out of five pigs measured *in vivo* presented with a lateral pneumothorax after instillation of air in the pleural space. The area of a pneumothorax could be easily identified as a non-scattering space between the lateral portion of the rib cage and the strongly scattering lung parenchyma in dark-field images. Even small pneumothoraces, which were occult in the corresponding transmission images, were visible in the dark-field images because of the high signal contrast between the lung parenchyma and the pleural cavity (Figs 1 and 2). Major pneumothoraces were visible in both dark-field and transmission images. A thin, sharp pleural line in the peripheral parts of the affected thoracic side marked the area of a pneumothorax in transmission images. The area lateral to this pleural line was more radiolucent than the lung parenchyma, indicating the presence of free air in the pleural cavity. In dark-field images, a non-scattering, dark area next to the scattering lung parenchyma indicated major pneumothoraces. However, the identification of pneumothoraces in transmission images was more challenging compared to the diagnosis in dark-field radiographs, mainly due to a rather small contrast difference between the inflated lung and the air-filled pleural space.

**Figure 3:**
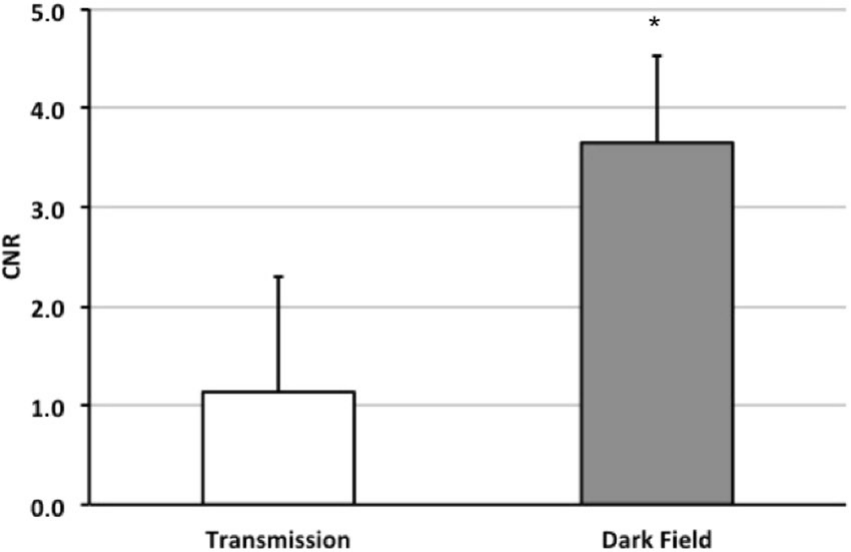
Contrast-to-noise ratio (CNR) between the air-filled pleural cavity and the adjacent lung tissue for transmission (white bar) and dark-field images (grey bar).CNR was significantly higher for dark-field compared to transmission imaging (*p* < 0.01).

Contrast-to-noise ratios were calculated to quantitatively evaluate the observations from qualitative image analysis of lateral pneumothoraces. When comparing lung-to-pneumothorax CNRs in the dark-field and transmission modalities (Fig. 3), a significantly higher CNR was measured in low-pass-filtered dark-field images (3.65 ± 0.9) than in unfiltered transmission images (1.13 ± 1.1) (*p* = 0.002). Several regions of interest determined for CNR analysis are shown in the Supplementary Material. CNR was 1.14 ± 1.1 for low-pass-filtered transmission images and 2.45 ± 0.7 for unfiltered dark-field images.

### Dorsal pneumothoraces – visual and quantitative analyses

**Figure 4:**
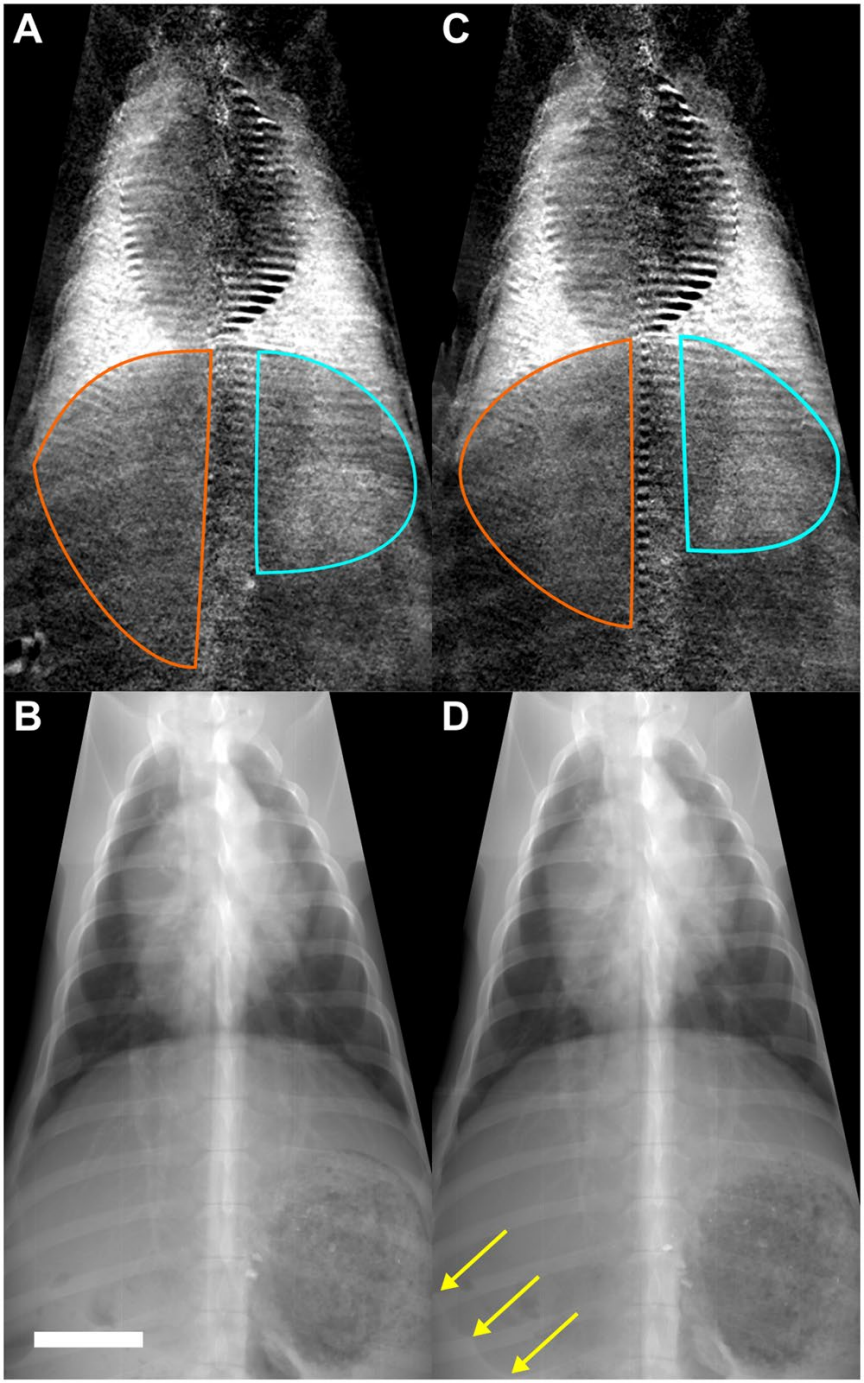
Depiction of a dorsal pneumothorax of a living pig in dark-field and transmission imaging.Scattering lung parenchyma can be visualized directly in dark-field images, even if located behind the diaphragm, making quantification of the area of the inflated lung possible. Before the induction of a pneumothorax (**A**,**B**) affected side (orange) 72 cm^2^, unaffected side (blue) 55 cm^2^. After the induction of a pneumothorax (right): affected side (orange) 61 cm^2^, unaffected side (blue) 56 cm^2^. Indirect signs, such as an increased hyper transparency in the basal parts of the affected lung and consecutively sharper edges of the pleura, indicate the presence of a pneumothorax in the corresponding transmission image (arrows). The white scale bar is approximately 5 cm.

For two lungs, a lateral pneumothorax could not be detected, although the pleural spaces were inflated with considerable amounts of air (50 and 150 ml). As the pigs were placed in the abdominal position, there is the change that the pleural line cannot be detected laterally, as the air in the pleural space accumulates dorsally, along the space that is located uppermost^20^, which – in this study setup – is the posterior basal space, located behind the diaphragm and the organs of the upper abdomen, causing the lung parenchyma in this area to collapse. When carefully analyzing the dark-field images before pneumothorax induction, the dorsal parts of the lower lung that are located behind the diaphragm showed a weak but still clearly visible dark-field signal. These sections of the lungs appeared considerably smaller after induction of a pneumothorax, indicating a partial collapse of the affected lung due to compression by pleural air located in the uppermost areas of the thorax. After quantifying the projected area of the lung located behind the diaphragm (which is possible as the scattering lung is directly and exclusively visible in the dark-field images), a significant decrease of inflated lung parenchyma was found after a unilateral pneumothorax was induced [mean area of the affected lung before the induction of a pneumothorax: (62.8 ± 7.8) cm^2^, afterwards: (50.0 ± 7.2) cm^2^; −20.5%; *p* < 0.0001; n = 8]. The area of the unaffected side was analyzed as an internal control, showing no significant difference in size before and after the induction of a pneumothorax [mean area of the affected lung before a pneumothorax was induced: (57.6 ± 10.3) cm^2^, afterwards: (57.0 ± 7.5) cm^2^; −0.3%; *p* = 0.9; n = 8]. In the corresponding transmission images, a slight increase in transparency with consecutively sharper margins of the basal pleural space was slightly visible, serving as a discrete, indirect sign for a pneumothorax, the so-called deep sulcus sign (Fig. 4)^20^.

## Discussion

Even initially small pneumothoraces have the potential to dramatically enlarge over a short period of time, making early and reliable diagnosis of this disease essential to prevent serious health consequences, such as a tension pneumothorax, which – if not detected and treated immediately – will result in cardiac failure^23^. Accurate diagnosis of a pneumothorax is affected by the low contrast between radiolucent lung parenchyma and the air-filled pleural space in conventional transmission images^24^. As chest radiography is the standard diagnostic tool for the detection of pneumothoraces, adding the information provided by dark-field images may significantly increase the diagnostic sensitivity.

The lung contains a myriad of air-tissue interfaces due to its alveolar microstructure. This leads to a pronounced coherent scatter of x-rays resulting in a strong dark-field signal, which significantly increases the contrast between the lung and the air-filled pleural space. A previous study in a murine model has demonstrated the detection of pneumothoraces to be markedly improved when analyzing dark-field compared to transmission images^7^. We now translated the experimental setup from a small animal to a large animal model in order to identify the potential diagnostic value of dark-field imaging in a setting similar to clinical reality. Using a prototype large-object x-ray dark-field scanner, it is now possible for the first time to depict lungs comparable in size to a human organ. In line with our hypothesis, we were able to show that diagnosing pneumothoraces can be facilitated using this new imaging method. Since the contrast-to-noise ratio between the area of a pneumothorax and the adjacent lung was significantly higher for the dark-field than for the transmission signal, identification of very small, lateral pneumothoraces was exclusively possible in dark-field images. Additionally, diagnosis of dorsal pneumothoraces was facilitated due to the high signal of the lung parenchyma, which was visible even if the basal parts of the lungs were located behind the organs of the upper abdomen. Especially when thinking of a potential clinical implementation of this imaging technique, facilitated diagnosis of dorsal or, if the images are acquired in supine position, ventral pneumothoraces is of special interest: Severely ill patients, e.g. those in intensive care, have a high risk of suffering from a pneumothorax because they need to undergo various interventions (such as placement of a central venous catheter or thoracentesis) that might result in a pneumothorax^25^. Supine chest x-rays are routinely ordered to exclude this complication^26^. As the sensitivity of diagnosing a ventral pneumothorax with conventional transmission imaging is limited, an imaging technique directly visualizing collapsed lung parenchyma is of great advantage. The deep sulcus sign, an indirect and rather unspecific sign for the presence of a ventral/dorsal pneumothorax, is difficult to detect compared to the direct visualization of lung parenchyma using x-ray dark-field imaging. As transmission and dark-field radiographs are generated at the same time, combining information from both imaging modalities (anatomic details such as margins of the pleural cavity in transmission images, collapse of lung parenchyma in dark-field images) is possible with this imaging set-up.

The results of the presented work need to be seen in light of the study design and its limitations. Because this study was designed as a feasibility study, only a small number of animals was used for the experiments. Due to animal welfare reasons, the number of experimental animals also needed to be limited to the utmost minimum.

In this study, *ex vivo* as well as *in vivo* animal setups were used to obtain dark-field and transmission images of pneumothoraces. For signal analysis, data of both animal groups was combined. This was possible as no statistically significant difference between *in vivo* and *ex vivo* measurements for transmission and dark-field CNRs was found. However, a clear distinction between animals measured *in vivo* and *ex vivo* was possible in dark-field images due to motion artifacts appearing as horizontal streaks located at the edges of the heart caused by the heartbeat (Supplementary Figure 1). These artifacts result from organs entering and/or exiting a given image region while the intensity fringe pattern is scanned over it. The intensity modulations from the movement are then misinterpreted during signal extraction as an intensity modulation due to fringe scanning. The artifact magnitude appears to depend on fringe pattern phase. The artifacts therefore present as horizontal lines (orthogonal to the scanning direction), similar to the spatial orientation of the fringe pattern.

The visualization of very small, lateral pneumothoraces was exclusively possible using dark-field imaging in our study setting. Because of the increased contrast-to-noise ratio between lung parenchyma and pneumothorax in dark-field images, even discrete pleural detachment was visible. As a control image was acquired prior to the induction of a pneumothorax, comparison of both images might lead to an increased visibility of the pneumothorax. To answer the question whether dark-field imaging can actually facilitate the diagnosis of small lateral pneumothoraces, further research, for example by implementation of a reader study, is required.

To calculate CNRs, data from low-pass-filtered dark-field images and unfiltered transmission images were used. Filtering was performed using a 2D Gaussian filter kernel with a FWHM of 3.25 pixels (1.1 mm in the object plane) in both dimensions. This was done to ameliorate the inferior noise characteristics of x-ray dark-field imaging (compared to regular radiography) and trade a CNR increase for a decrease in spatial resolution. Using this filter, very small features (of the order of the filter FWHM) become undetectable, but other features (such as the examined pneumothoraces) are delineated more clearly. This was deemed acceptable because no significant features could be discerned in the unfiltered dark-field images that were lost due to filtering. The filter was not considered useful for the transmission modality because it improved the CNR only marginally. To optimize overall image impression, a somewhat different set of post-processing filters was used for the dark-field images in Figs 1, 2, and 4: A selective Fourier-based filter was used to detect and low-pass-filter areas with grating tile gaps, and a 3 × 3 binomial filter kernel was successively applied to the whole image. The resulting images were not used for quantitative analysis.

When calculating the CNR for filtered transmission images (1.14 ± 1.1), CNR for filtered dark-field images still was significantly higher (*p* = 0.002). The same was found when comparing the CNR for unfiltered dark-field images (2.45 ± 0.7) to that of unfiltered transmission images (*p* = 0.03).

We emphasize that the measured standard deviations are (for both modalities) a combination of photon noise and “anatomical noise” (actual variation of signal levels within the ROI due to unresolvable lung structure, i.e. not a truly random effect, see e.g. ref.^27^). It is challenging to separate both effects from the limited number of scans. The fact that CNR values are hardly increased by low-pass filtering suggests that they are limited by anatomical noise (of low spatial frequency). This would also suggest that detector dose increase only provides marginal improvements in the attenuation modality: Excluding changes in spatial resolution, the applied filter should be equivalent to a 25-fold dose increase (five-fold CNR increase) in areas where pixel values are uncorrelated and normally distributed with equal mean and variance.

For the dark-field modality, the CNR increase is greater, but this may also partly be due to anatomical noise: the high granularity of lung parenchyma may lead to a variation of dark-field signal levels of high spatial frequency. This is currently indistinguishable from photon noise, and would be attenuated more strongly by filtering than low-frequent anatomical noise in the transmission image. Experience from pathologies in small animal models leads us to believe that such a high-frequency signal variation has limited diagnostic value, i.e., some low-pass filtering probably does not affect the diagnostic value of dark-field images.

Determining the true spatial dark-field signal variation within the lung would require e.g. a time series of perfectly registered scans. This is not possible with the presented imaging protocol, since ventilation may only be stopped for one scan at a time and ventilation pressure during breath stop cannot be replicated perfectly, limiting registration quality.

Since no structures are clearly recognizable within each ROI, anatomical noise appears to affect image quality in the same fashion as true noise. We therefore believe that the combination of both noise sources presents a reasonable measure for quantifying the discriminability of pneumothorax and lung parenchyma.

CT scans of the lungs served as the standard of reference in this study to prove the presence of a pneumothorax. The scans can however not be used to draw a conclusion about the size of the pneumothoraces. As the CT scanner was not located in the same facility as the large-animal x-ray dark-field scanner, the pigs had to be disconnected from the mechanical respirator. Although the animals stayed intubated and the aboral part of the laryngeal tube as well as the thoracic puncture site were carefully sealed, an enlargement of the pneumothoraces during transport could not be ruled out.

Before dark-field imaging of the lung can be used in a clinical setting, further technical developments are required. The image acquisition time for this setup is 30–40 seconds. Especially for elderly people or patients with impaired lung function, breath-hold times of 30 seconds are often unachievable^28^. To avoid breathing artifacts, an image acquisition time of five seconds should not be exceeded. The dose area product (DAP) in our study was determined to be 0.5 Gy cm^2^. Assuming a tissue weighting factor of K = 0.16 (equivalent to a human lung^29^), this would result in an organ-specific dose of 0.08 mSv. This radiation dose is approximately six times higher than in a conventional p.a. chest x-ray^30^ and is acceptable given the additional information provided by the dark-field images. In conventional chest radiography, tube voltages of up to 125 kVp are standardly used^30^. In order to evaluate whether the tube voltage can be changed from 70 kVp – as used in our experimental setup – to higher values while preserving dark-field images, further research is necessary. To simultaneously achieve high visibilities and a significant dark-field signal at x-ray energies far beyond 70 kVp, or to improve image quality for currently-used tube voltages, gratings with small periods (to achieve high sensitivity) and high absorber heights (to achieve high visibilities) are required. Achieving such high aspect ratios may require further advances in grating development.

The optimal trade-off between resolution and dark-field signal levels should also be evaluated: although spatial resolution could be increased by reducing pixel size (e.g., disabling binning), we believe that the associated noise increase may not be desirable from a diagnostic point of view.

This feasibility study is an important step towards future clinical implementation of the x-ray dark-field imaging technique. It was not only possible to visualize lungs comparable to the size of human organs, but the diagnosis of pneumothoraces was also facilitated by the addition of information from dark-field images. Prospectively, this may offer the opportunity to improve clinical care for patients suffering from pneumothoraces.

## Materials and Methods

### Large animal protocol

All animal procedures were performed with permission of the local regulatory authority, Regierung von Oberbayern (ROB), Sachgebiet 54, 80534 München, approval number AZ 55.2-1-54-2532-61-2015. The ethics committee reviewed the application according to §15 TSchG German Animal Welfare Law.

German Landrace Hybrid pigs [wildtype, Chair for Molecular Animal Breeding and Biotechnology, Ludwig-Maximilians-University Munich breeding facility; n = 8 (*ex vivo* n = 3; *in vivo* n = 5), weight = (25.8 ± 5.9) kg; age 2.5–3.0 months, anterior-posterior thoracic diameter (20.2 ± 2.3) cm] served as experimental animals in this study. Pigs measured *ex vivo* were euthanized immediately before intubation and image acquisition. Pigs measured *in vivo* were sedated by intramuscular application of ketamine (Ursotamin®, Serumwerk Bernburg, Germany, 20 mg/kg) and azaperone (Stresnil®, Elanco Animal Health, Bad Homburg, Germany, 2 mg/kg). Anesthesia was accomplished by an initial intravenous bolus injection of propofol (Propofol 2%, MCT Fresenius, Fresenius Kabi, Langenhagen, Germany) and continued using a syringe pump (Injectomat® MC Agilia, Fresenius Kabi, Langenhagen, Germany) for permanent application of propofol, quantitatively adjusted to the animals’ individual needs. Heart frequency and oxygen saturation were monitored throughout the whole experiment using a pulse oximeter (Palmcare Plus, Medical Econet, Oberhausen, Germany). Both pigs measured *ex vivo* and *in vivo* were intubated and kept under automated breathing using a mechanical respirator (Fabius® Plus 2, Draeger, Luebeck, Germany) throughout the experiment. Mechanical ventilation parameters were set to: respiratory rate 12 breaths/min, breath volume 15 ml/kg body weight, peak airway pressure 22 mbar, PEEP (positive end-expiratory pressure) 2 mbar. In order to acquire dark-field and transmission images of the animals’ lungs, breathing was stopped for the duration of the measurements (approximately 30 seconds) with a constant airway pressure of 12 mbar. As the pigs were placed in an abdominal position all images were acquired in posterior-anterior (p.a.) direction. The pigs’ lungs were imaged before and immediately after induction of a unilateral pneumothorax. Pneumothoraces were induced 10 minutes after intravenous application of 0.05 mg fentanyl for analgesia. The skin was incised at the level of the anterior axillary line, between the fifth and seventh intercostal space. The correct intercostal space was identified using sonography. Subsequently, a thin catheter (Central Venous Catheterization Set with Blue FlexTip Catheter, Arrow International, Morrisville, North Carolina, USA) was placed in the pleural cavity. A Heidelberger elongation and a three-way stopcock were attached to the catheter and the pleural space was insufflated with 50–500 ml air using a 50 ml perfusion syringe (Perfusion Syringe, Becton, Dickinson and Company, Franklin Lakes, New Jersey, USA) to generate variously sized pneumothoraces^31^. After image acquisition, the animals measured *in vivo* were sacrificed under anesthesia by intravenous injection of 0.1 ml/kg body weight T61^®^ (Intervet GmbH, Unterschleissheim, Germany). Subsequently, a computed tomography (CT) scan (GE Discovery CT750, General Electrics Healthcare, USA) of all animals was performed to serve as the standard of reference for the induction of the pneumothorax.

### Imaging protocol

All images were acquired using a prototype large-object chest-x-ray scanner described previously^8^. The scanner’s grating arrangement consists of three gratings: a source grating (G0, area: 5.0 × 2.5 cm^2^, period: 68.72 μm, duty cycle: 0.7), a reference grating (G1, period: 8.73 μm, duty cycle: 0.5) and an analyzer grating (G2, period: 10 μm, duty cycle: 0.5). The G1 and G2 gratings each consist of eight linearly arranged half-tiles of 5 × 2.5 cm^2^ and were tiled using the procedure described in ref.^32^. All gratings are absorption gratings with gold filling (gold heights range from 150 to 200 μm). They were manufactured by KIT (G2) and Microworks (G0, G1) using the LIGA process^33^. The distance between source and detector is 2 m. The G0-G1 and G1-G2 distances are 1.60 and 0.25 m, respectively. This results in an arrangement previously presented in ref.^34^. It differs from a Talbot-Lau setup in that the G2 grating is placed so close behind the G1 grating that it analyses not a Talbot self-image, but the direct shadow of the G1 grating. Direct use of these “beamlets” is a feature shared with the edge illumination technique^35^, which may also be used for X-ray scatter imaging^36^. The sample table is located 5.6 cm upstream of the G1 grating. The x-ray source (Philips SRO 1750 ROT 360, Philips Medical Systems, Hamburg, Germany) with a Tungsten rotating anode is operated at 70 kVp, 450 mA tube current and 12 Hz pulse frequency. A Pixium RF4343 (Trixell, Moirans, France) flat-panel detector was used in 3 × 3 binning mode, resulting in a true pixel size of 444 μm and an effective pixel size of 360 μm. The field-of-view in the object plane is 32 × 35 cm^2^.

### Quantitative image analysis

To quantify dark-field and transmission signal intensities in the area of the pneumothorax and the adjacent lung, regions of interest (ROIs) were defined. The ROIs were placed in the air-filled pleural space and in the adjacent lung parenchyma; care was taken to exclude the rib cage as well as mediastinal structures. Identical ROIs were used for transmission and dark-field images. As transmission and dark-field images result from the same exposure, they are perfectly registered. The contrast-to-noise ratio (CNR) between the air-filled pleural cavity and the adjacent lung tissue was calculated for dark-field and transmission images according to the equation

$${\rm{CNR}}=\frac{|{\mu }_{{\rm{L}}}-{\mu }_{{\rm{P}}}|}{\sqrt{{\sigma }_{{\rm{L}}}^{{\rm{2}}}+{\sigma }_{{\rm{P}}}^{{\rm{2}}}}},$$

where μ_L_ and μ_P_ represent the mean values of the signals over the lung and pneumothorax ROI, respectively, and σ_L_ and σ_P_ are the corresponding standard deviations. To quantify dorsal pneumothoraces, the projected area in the object plane of the lung located behind the diaphragm was identified by placing an ROI around the scattering lung before and after a unilateral pneumothorax was induced. The same was done for the side of the lung that was not affected by a pneumothorax, therefore serving as an internal control.

### Statistical analysis

Means and standard deviations for lung sizes and signal intensities from dark-field and transmission images were calculated. Results were tested for statistical significance by using Student’s two-tailed t test for paired samples. For the sample size of n = 6, a power of 1 − β = 99.9% was calculated for a mean paired difference of μ_d_ = 1.933 with a standard deviation of σ_d_ = 0.734 and a level of significance of α = 5%. MedCalc® (version 14.12.0, Mariakerke, Belgium) was used for all statistical calculations.

## Electronic supplementary material

Supplementary Material
