# Supplementary material for: Depiction of pneumothoraces in a large animal model using x-ray dark-field radiography
Source: Sci Rep. 2018 Feb 8;8:2602. doi: 10.1038/s41598-018-20985-y (PMC5805747; doi:10.1038/s41598-018-20985-y)
Supplement: Supplementary file 1 — Supplementary Material [file 41598_2018_20985_MOESM1_ESM.pdf]

Depiction of pneumothoraces in a large animal model using x-ray dark-field radiography

Authors:

Katharina Hellbach[1,*], Andrea Baehr[2,*], Fabio De Marco[3], Konstantin Willer[3], Lukas Gromann[3], Julia Herzen[3], Michaela Dmochewitz[2], Sigrid Auweter[1], Alexander A. Fingerle[4], Peter B. Noël[3,4], Ernst J. Rummeny[4], Andre Yaroshenko[5], Hanns-Ingo Maack[5], Thomas Pralow[5], Hendrik van der Heijden[5], Nataly Wieberneit[5], Roland Proksa[6], Thomas Koehler[6], Karsten Rindt[5], Tobias J. Schroeter[8], Juergen Mohr[8], Fabian Bamberg[1,9], Birgit Ertl-Wagner[1,9], Franz Pfeiffer[3,4] & Maximilian F. Reiser[1,9]

Affiliations:

[1] Department of Radiology, University Hospital, LMU Munich, 81377 Munich, Germany.
[2] Chair for Molecular Animal Breeding and Biotechnology, Ludwig-Maximilians-University Munich, 85764 Oberschleißheim, Germany.
[3] Chair of Biomedical Physics & Munich School of BioEngineering, Technical University of Munich, 85748 Garching, Germany.
[4] Department of Diagnostic and Interventional Radiology, Technical University of Munich, 81675 Munich, Germany.
[5] Philips Medical Systems DMC GmbH, 22335 Hamburg, Germany.
[6] Philips GmbH Innovative Technologies, Research Laboratories, 22335 Hamburg, Germany.
[7] Institute for Advanced Study, Technical University of Munich, 85748 Garching, Germany.
[8] Institute of Microstructure Technology, Karlsruhe Institute of Technology (KIT), , 76344 Eggenstein-Leopoldshafen, Germany.
[9] German Center for Lung Research (DZL), Comprehensive Pneumology Center (CPC-M), Helmholtz Zentrum Munich, 81377 Munich, Germany.

* These authors contributed equally.

Corresponding Author:

Katharina Hellbach, MD
Department of Radiology
University Hospital, LMU Munich
Marchioninistr. 15
81377 Munich, Germany.
Tel +49.89.4400.73620
Fax +49.89.4400.78832
Email: katharina.hellbach@med.uni-muenchen.de

Supplementary Material

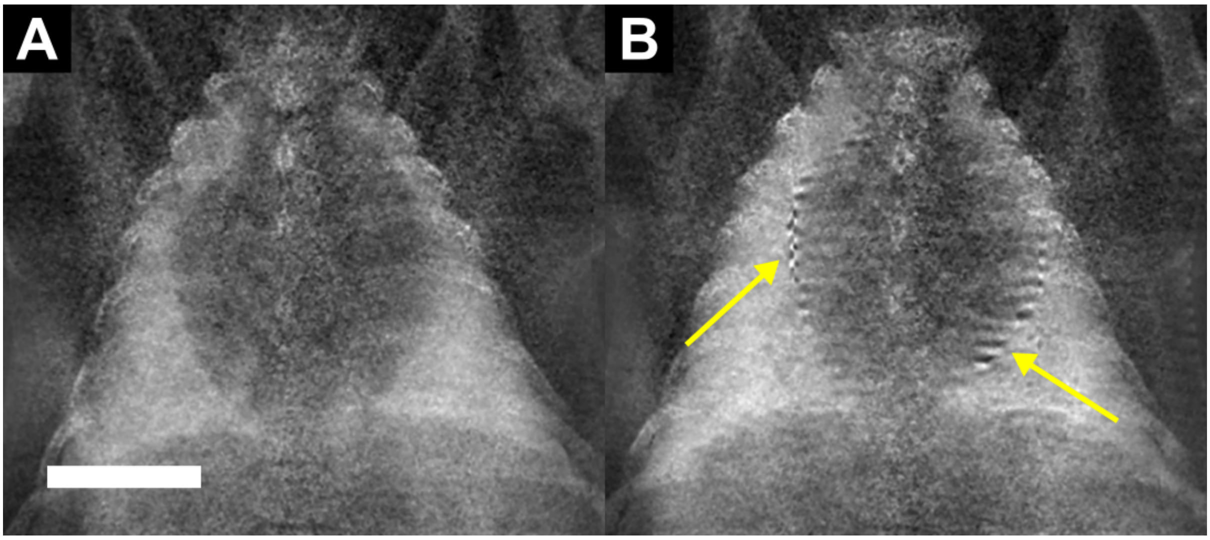

Supplementary Figure 1. Comparison of pigs measured *ex vivo* versus *in vivo* in dark-field images. As indicated by the black arrow, the main difference enabling a distinction between dark-field images acquired *ex vivo* (A) and *in vivo* (B) are the motion artifacts due to the heartbeat). The white scale bar is approximately 5 cm.

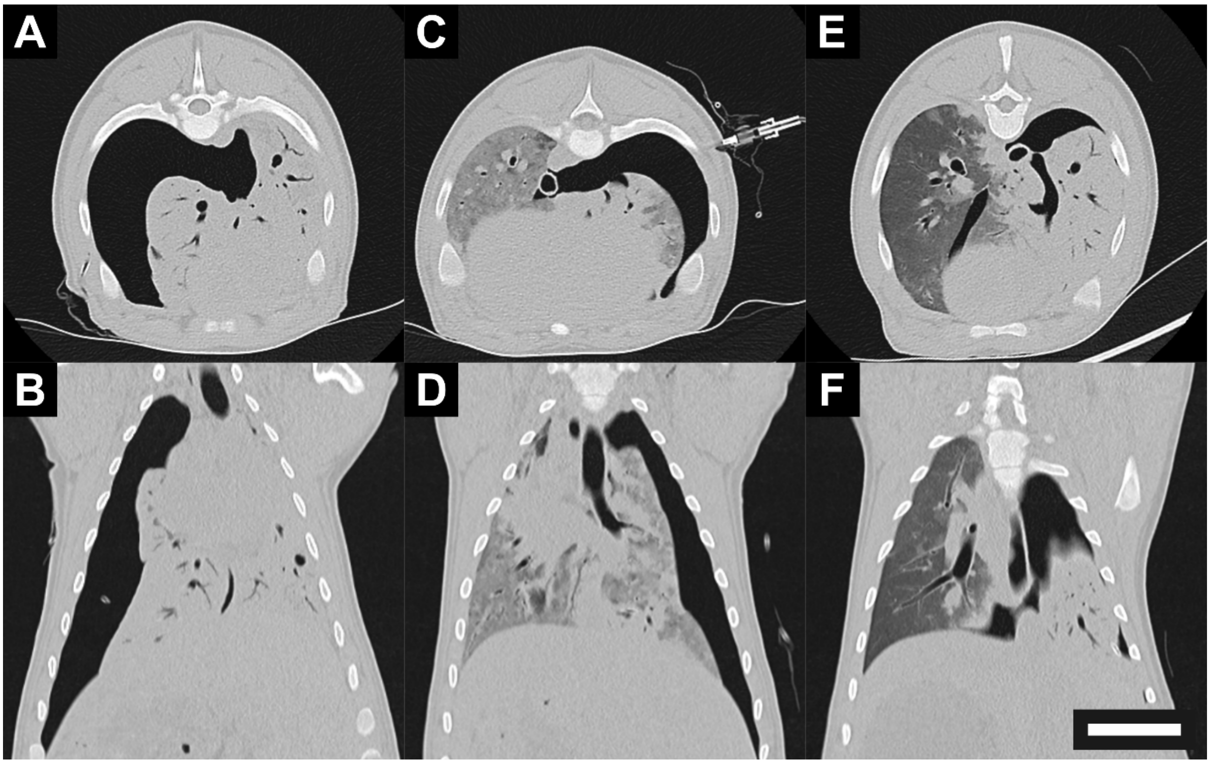

Supplementary Figure 2. Transverse (top row) and coronal (bottom row) CT slices of three pigs after pneumothorax induction. The animals were euthanized prior to CT imaging due to the necessary transport. The catheter used for pneumothorax induction is visible in Figure C. Slice thickness: 2.5 mm (transverse) / 3.0 mm (coronal), voxel size: 0.51 mm (A,B) / 0.56 mm (C,D) / 0.47 mm (E,F). Window/Level: 1685 / -335 HU. The white scale bar is approximately 5 cm.

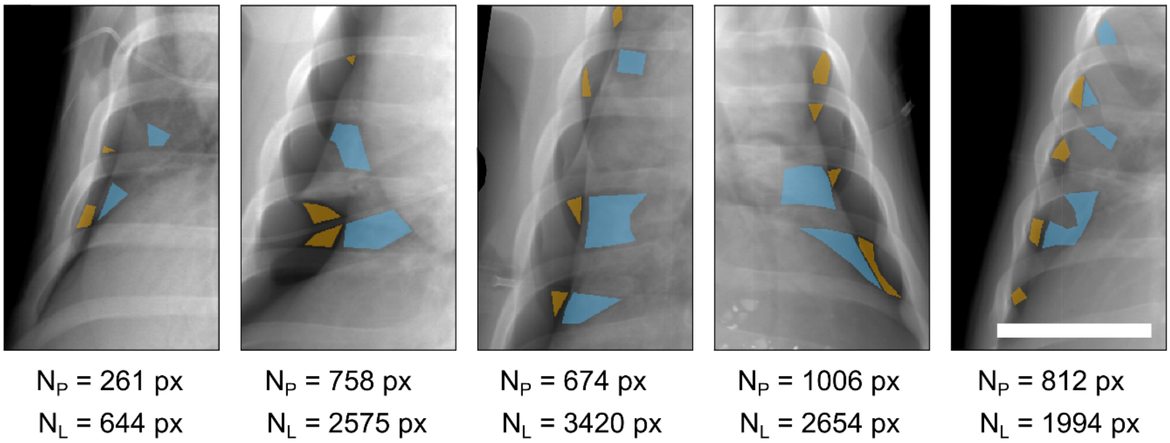

Supplementary Figure 3. Example ROIs selected for the calculation of CNR values, superimposed onto transmission images. Regions containing lung parenchyma are shown in blue, pneumothorax regions are shown in orange. All ROI sections are manually drawn polygons defined with scripts using the Python programming language (Python Software Foundation, https://www.python.org) and associated scientific programming libraries. Number of pixels in ROIs are given (N_L: lung, N_P: pneumothorax). Care was taken to exclude ribs from the selected regions. The white scale bar is approximately 5 cm.

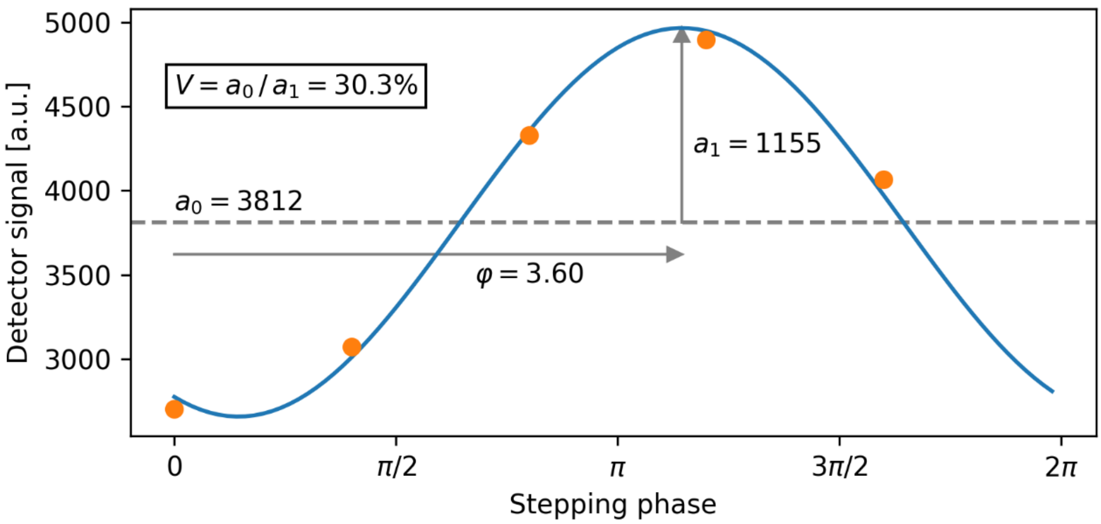

Supplementary Figure 4. Stepping curve from a blank scan with fitted sinusoidal intensity model. Acquisition parameters: 70 kVp, 50 mA, 5 phase steps over one fringe period, 20 ms exposure per phase step.
